# A Novel Synthetic Microtubule Inhibitor, MPT0B214 Exhibits Antitumor Activity in Human Tumor Cells through Mitochondria-Dependent Intrinsic Pathway

**DOI:** 10.1371/journal.pone.0058953

**Published:** 2013-03-12

**Authors:** Nai-Jung Chiang, Ching-I Lin, Jing-Ping Liou, Ching-Chuan Kuo, Chi-Yen Chang, Li-Tzong Chen, Jang-Yang Chang

**Affiliations:** 1 National Institute of Cancer Research, National Health Research Institutes, Tainan, Taiwan, ROC; 2 Division of Hematology/Oncology, Department of Internal Medicine, National Cheng Kung University Hospital, Tainan, Taiwan, ROC; 3 Division of Hematology/Oncology, Department of Internal Medicine, St. Martin De Porres Hospital, Chiayi, Taiwan, ROC; 4 School of Pharmacy, College of Pharmacy, Taipei Medical University, Taipei, Taiwan, ROC; Innsbruck Medical University, Austria

## Abstract

Agents that interfere with mitotic progression by disturbing microtubule dynamics are commonly used for cancer treatment. Previously, a series of aroylquinolone regioisomers as novel microtubule inhibitors were discovered. One of these new compounds, MPT0B214 inhibited tubulin polymerization through strongly binding to the tubulin’s colchicine-binding site and had cytotoxic activity in a variety of human tumor cell lines. After treatment with MPT0B214, KB cells were arrested in the G_2_-M phase before cell death occurred, which were associated with upregulation of cyclin B1, dephosphorylation of Cdc2, phosphorylation of Cdc25C and elevated expression of the mitotic marker MPM-2. Furthermore, the compound induced apoptotic cell death through mitochondria/caspase 9-dependent pathway. Notably, several KB-derived multidrug-resistant cancer cell lines were also sensitive to MPT0B214 treatment. These findings showed that MPT0B214 is a potential compound in the treatment of various malignancies.

## Introduction

Tubulin-containing structures, such as microtubules, are important for the formation of the mitotic spindle during mitosis. Microtubules consisting of alpha- and beta- tubulin heterodimers are responsible for various fundamental cell functions, including sustained shapes, chemotaxis, the intracellular transport of organelles and vesicles, and the regulation of motility [1], [2]. Tubulin binding molecules that disrupt microtubules can lead to cell cycle arrest in the M phase, forming abnormal mitotic spindles and triggering programmed cell death [3]. Although natural microtubule-targeting drugs such as taxanes and vinca alkaloids have been used successfully in clinical use, they carry substantial limitations, such as intrinsic or acquired drug resistance, serious side effects, complex syntheses, and difficulties in isolation procedures [4], [5]. Among these mechanisms of drug resistance, the development of multidrug resistance (MDR) cannot be ignored. MDR is a multifactorial process that involves the overexpression of drug efflux pumps, such as P-glycoprotein (P-gp170/MDR) and multidrug resistance-associated proteins (MRPs). These efflux pumps have the ability to reduce the intracellular concentrations of drugs [6], [7]. Therefore, discovering microtubule-disrupting candidates that may overcome MDR and improve efficacy, tolerance toxicities, and pharmacokinetic profiles is pressing [8], [9], [10], [11].

**Figure 1:**
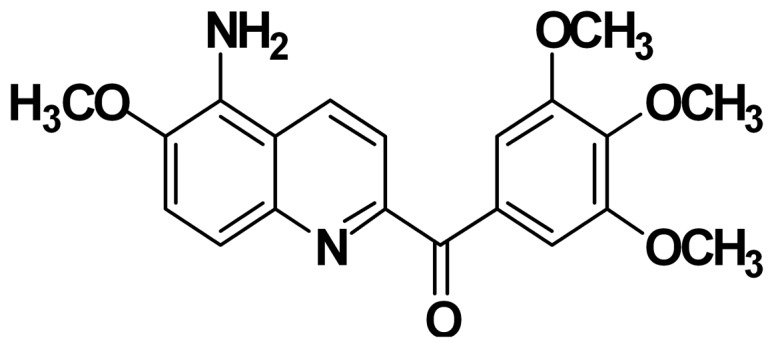
Chemical structure of 5-amino-6-methoxy-2-(3′,4′,5′-trimethoxybenzoyl)-quinoline (MPT0B214).

Analysis of these microtubule inhibitors, such as combretastatin A-4 (CA-4), AVE-8062, colchicine, and ABT-751, indicates that 3,4,5-trimethoxyphenyl/3,4,5-trimethoxybenzoyl and para-mehoxyphenyl groups play an important role in their bioactivity. In addition, quinoline is a pharmacological class of heterocyclic compounds. We have attempted to synthesize a new class of compounds using a quinoline core coupled with the 3,4,5-trimethoxybenzoyl group as tubulin polymerization inhibitors. Studies of aroylquinoline regioisomers led to the discovery of 5-amino-2-aroylquinolines as novel highly potent inhibitors of tubulin polymerization [12]. One of these compounds, MPT0B214 (Fig. 1) showed potent anti-proliferative activity against tumor cell growth. We investigated the molecular targets, anticancer cytotoxic mechanisms of MPT0B214, and determined its effects on drug-resistant tumor cells.

## Materials and Methods

### Synthesis of MPT0B214

The compound, MPT0B214, was synthesized by Dr. Jing-Ping Liou at the College of Pharmacy, Taipei Medical University, Taipei, Taiwan, ROC. MPT0B214 is a yellow solid derived from the quinoline core and 3,4,5-trimethoxybenzoyl group. The synthetic methods and structures have been published [12].

### Chemicals and Reagents

Colchicine, paclitaxel, and vincristine were purchased from Sigma Chemical Co. (St. Louis, Mo). Media and reagent for cell culture fluorescents were acquired from Invitrogen (Carlsbad, CA). Microtubule-associated protein (MAP)-rich tubulin and biotin-labeled tubulin were obtained from Cytoskeleton, Inc. (Denver, CO). [^3^H]colchicine, [^3^H]paclitaxel, [^3^H]vinblastine, and streptavidin-labeled poly(vinyl toluene) scintillation proximity assay (SPA) beads were obtained from PerkinElmer Life and Analytical Sciences (Boston, MA) and Amersham Pharmacia Biotech (Piscataway, NJ), respectively. All other chemicals were from Sigma Chemical or Merck Co. (Darmstadt, Germany) with standard analytical or higher grade.

### Cell Culture

Human cervical carcinoma KB cells (this cell line was believed to be derived from an epidermal carcinoma of the mouth but now has been shown with HeLa characteristics), nasopharyngeal carcinoma HONE-1 cells, colorectal carcinoma HT-29 cells, non-small cell lung cancer H460 cells, and gastric MKN-45 were maintained in an RPMI 1640 medium supplied with 5% fetal bovine serum. Human breast cancer MCF-7 and gastric carcinoma TSGH cells were cultured with a minimal essential medium (MEM) supplied with 5% fetal bovine serum. Human normal lung WI-38 cells were maintained in α-MEM with 10% fetal bovine serum. The KB, HONE-1, HT-29, H460 and MCF-7 cell lines were purchased from American Type Culture Collection (Manassas, VA), and MKN-45 and TSGH were purchased from the Health Science Research Resources Bank (Osaka, Japan). The vincristine-resistant cell line KB-VIN10, etoposide-resistant cell line KB-7D, and paclitaxel-resistant cell line KB-S15 were maintained in a growth medium supplemented with 10 nM vincristine, 7 μM etoposide, and 50 nM paclitaxel, respectively. KB-VIN10 and KB-S15 overexpressed P-gp170/MDR. KB-7D displayed downregulation of topoisomerase II and overexpression of MRP [13], [14].

### Growth Inhibition Assay

Cells in the logarithmic growth phase were cultured at a density of 5000 cells/ml/well in a 24-well plate. The resistant cell lines were kept in a drug-free medium for 3 d before the experiments. The cells were exposed to various concentrations of the tested drug for 3 generation times. Methylene blue dye was used to evaluate the effects of the test drugs on cell growth and to determine the concentration of drugs that inhibited 50% of cell growth [15].

### Cell Cycle Analysis

Cell-cycle progression was monitored using DNA flow cytometry. KB cells were cultured in 10 cm tissue culture dishes until confluence was 80% and were treated with 10 nM MPT0B214 for the indicated time. Cellular DNA was stained with PBS containing 50 μg/ml propidium iodide (PI) and 50 μg/ml RNase in the dark at room temperature for 20 min. The cell cycle was determined by a fluorescence-activated cell sorting IV flow cytometer (BD Biosciences, Franklin Lakes, NJ) with 10000 cells scored. Data were analyzed and graphs were prepared using the Modfit 2.0 program (Verity Software House, Topsham, ME).

### Western Blot Analysis

Cell lysates were prepared using an ice-cold lysis buffer (50 mM Tris, pH 7.4, 0.8 M NaCl, 5 mM MgCl_2_, 0.5% NP-40, 1 mM phenylmethylsulfonyl fluoride, and 20 μg/ml each of proteinase inhibitor cocktail: aprotinin, leupeptin, and pepstatin). Lysates were centrifuged at 12000 g for 15 min, and the supernatants were collected and quantified. Equal amounts of lysate (on a protein basis) were then differentiated by SDS-PAGE, and transferred onto PVDF membranes. The primary antibodies against various proteins were obtained from the following sources: α-tubulin (Sigma), Bcl2, phospho-Cdc2, Cdc25C, cyclin B1, and FAS (Santa Cruz Biotechnology, Inc., Santa Cruz, CA); phosphospecific MPM-2 (Upstate Biotechnology, Lake Placid, NY); COX IV (Cell signaling Technology Inc., Beverly, MA); poly(ADP-ribose) polymerase (PARP) (Severigen, Gaithersburg, MD); FasL (Oncogene Research Products, Darmstadt, Germany). The secondary antibodies of goat anti-rabbit IgG-horseradish peroxidase (HRP) conjugates and goat anti-mouse IgG-HRP conjugates were purchased from Santa Cruz Biotechnology, Inc. (Santa Cruz, CA).

### Immunocytochemistry

KB cells plated on coverslips were treated with indicated concentrations of the tested compound for 6 h. After treatment, cells were fixed in methanol/acetone (1∶1 v/v) at −20°C for 1 h and then washed with PBS. Subsequently, the cells were blocked with 5% skim milk in PBS for 1 h and further incubated with 5% skim milk containing anti-α-tubulin monoclonal antibody (1∶500 dilutions) for 2 h at room temperature. Cells were washed with PBS twice to remove excess antibodies and then probed with an FITC-conjugated secondary antibody (1∶200 dilution) for 1 h at room temperature. The images of cellular microtubules were captured with an Olympus BX50 fluorescence microscope (Dulles, VA). For comparison, we also included paclitaxel and colchicine, along with MPT0B214.

### Tubulin Competition-binding Scintillation Proximity Assay (SPA)

In brief, [^3^H]colchicine, [^3^H]paclitaxel, or [^3^H]vinblastine sulfate with final concentrations of 0.08, 0.16, or 0.25 μM, respectively, were mixed with the tested compound and special long chain biotin-labeled tubulin (0.5 μg for colchicine binding assay and paclitaxel binding assay, and 1.0 μg for the vinblastine binding assay) in a buffer of 100 μl containing 80 mM PIPES (pH 6.8), 1 mM EGTA, 10% glycerol, 1 mM MgCl2, and 1 mM GTP for 2 h at 37°C. Streptavidin-labeled poly (vinyl toluene) SPA beads (80, 160, and 200 μg for colchicines, paclitaxel, or vinblastine binding assays, respectively) were then added to each reaction mixture. The radioactive counts were then directly measured by a scintillation counter, and the inhibition constant (*K_i_*) was calculated using the Cheng-Prusoff equation [16].

### *In vitro* Microtubule Assembly Assay

The assay was performed according to Bollag et al. [17]. In a 96-well microtiter plate, 0.24 mg of microtubule-associated protein-rich tubulin was mixed with various concentrations of test compounds and incubated at 37°C in a 120 ml reaction buffer (100 mM PIPES, pH 6.9, 1.5 mM MgCl_2_, 1 mM GTP and 1% (v/v) DMSO). A350 nm was monitored at 37°C and recorded every 30 s for 25 min using PowerWave X Microplate Reader (BIO-TEK Instruments, Winooski, VT). The augmentation in A350 nm indicated the increase in tubulin polymerization. The area under the curve of the untreated control was set to 100% polymerization, and IC_50_ was calculated using nonlinear regression.

### *In vivo* Microtubule Assembly Assay

Separation of polymerized tubulin from tubulin dimmers and analysis of the effect of MPT0B214 on tubulin polymerization in vivo were performed by Blagosklommy et al. [18]. KB and KB-VIN10 cells at a density of 1×10^6^/100 mm^2^ dishes were treated with indicated concentrations of test agents for the selected treatment duration. Cells were then washed with PBS 3 times before adding the lysis buffer containing 20 mM Tris-HCl, pH 6.8, 1 mM MgCl_2_, 2 mM EGTA, 1 mM PMSF, 1 mM orthovanadate, 0.5% NP-40, and 20 μg/ml proteinase inhibitor aprotinin, leupeptin, and pepstatin. Supernatants were collected after centrifugation at 15000 rpm for 10 min at 4°C. These yielded soluble tubulin dimers in the supernatant and polymerized microtubules in the pellet. The pellets were dissolved in an SDS-PAGE sampling loading dye and heated at 95°C for 10 min to dissolve the pellets and subjected to electrophoresis on 10% SDS-PAGE. After electrophoresis, the proteins were transferred to a nitrocellulose membrane, and then blocked with 5% skim milk/PBS-Tween20 overnight at 4°C. Immunoblots were probed with α-tubulin monoclonal antibodies and secondary HRP-conjugated antibodies. Detection of an immunoreactive signal was accomplished with a Western blot chemiluminescent reagent.

### Annexin V and Propidium Iodide (PI) Binding Assay

The early and late stages of apoptosis were monitored by annexin V-fluorescein (apoptotic cell marker) and PI (necrotic cell marker) double staining. The staining method was used according to the manufacturer’s staining kit (Roche Diagnostics, Mannheim, Germany). Control cells stained with Annexin-V or PI alone was used to compensate for flow cytometry analysis (BD Biosciences). Viable cells were those with both Annexin-V and PI-double negative staining. Annexin-V-positive and PI-negative cells were defined as early apoptotic cells, and Annexin-V and PI-double-positive cells were defined as late-arising apoptotic cells.

### Assays for Caspases Activities

Caspase activities were determined using Caspase Fluorometric Assay Kits (R&D Systems Inc., Minneapolis, MN) or the CaspACE Assay System Fluorometric (Promega Corporation, Madison, WI). Briefly, 1×10^6^ cells were treated with 10 nM of MPT0B214 and then incubated for different periods to determine the activities of caspase-3, caspase-8, and caspase-9, which were assessed according to the manufacturer’s instructions. Cells were harvested and lysed in a lysis buffer for 10 min. After centrifugation, an equal amount of the extract proteins were incubated with fluorescent substrates, which include Ac-DEVD-AMC, caspase 3 substrate; IETD-AFC, caspase 8 substrate; LEHD-AFC, and caspase 9 substrate in a reaction buffer. After incubation in a 96-well flat bottom microplate at 37°C for 1 h, the fluorescence of the cleaved substrate was determined using the Victor 1420 Multilable Counter (Wallac, Turku, Finland). The results are from 3 independent experiments.

### Mitochondrial Transmembrane Potential

Cells were initially seeded at 1×10^6^ in 100 mm^2^ dishes and then treated with various concentrations of MPT0B214 for 24 h. After drug treatment, cells were loaded with the probe DiOC_6_ (20 nM) for 30 min at 37°C before cytometric analysis. The cells were harvested and resuspended in PBS. Measurement of the retained DiOC_6_ in 10000 cells of each sample was performed in a FACSVantage flow cytometer (Becton Dickinson). DiOC_6_ was excited at 488 nm, and fluorescence was analyzed at 525 nm (FL-1) after logarithmic amplification.

### Preparation of Mitochondrial and Cytosolic Fractions

Cells were treated with MPT0B214 for the indicated periods. The mitochondria isolation kit (FOCUS™, Biosciences G) was used according to the manufacturer’s instructions to obtain the mitochondrial and cytosolic fractions. Cytochrome *c* was detected by a monoclonal antibody at a dilution recommended by the manufacturer. Tubulin and COX IV were probed as the internal control of the cytosolic and mitochondrial fractions, respectively.

### Statistical Analysis

All assays were repeated in triplicate, and data were expressed as means with standard deviations. The Student *t* test was used to compare the mean of each group with the control group. Differences with *p* values <0.05 were considered statistically significant.

## Results

### Growth Inhibition of MPT0B214 against Human Solid Cancer Cell Lines and Resistant Cell Lines with Over-expression of P-gp170/MDR and MRP

The anti-proliferative effect of MPT0B214 against various human cancer cell lines was determined by methyl blue assay, including cervical carcinoma KB cells, nasopharyngeal carcinoma HONE-1 cells, colorectal carcinoma HT29 cells, breast carcinoma MCF-7 cells, lung cancer H460 cells, gastric carcinoma TSGH, and MKN-45 cells, with IC_50_ values ranging between 5 and 15 nM (Table 1). We further examined the efficacy of MPT0B214 against drug-resistant cell lines, as shown in Table 2. KB-VIN10 cells have profound resistance to paclitaxel (3460-fold) and vincristine (150-fold) compared to parental cells. KB-S15 and KB-7D cells are mild to moderately resistant to paclitaxel and vincristine (2- to 30-fold). In contrast, MPT0B214 showed similarly potent cytotoxic efficacy between parental cells and those of KB-derived resistant cell lines with an IC_50_ of 4–6 nM. However, the IC_50_ of MPT0B214 toward normal human lung WI-38 cells was >1000 nM. This result demonstrated that MPT0B214 exhibits different drug sensitivity between WI-38 and other cancer cells.

**Table 1:** Growth inhibition of MPT0B214 against various human cancer cell lines.

Cell Line	Origin	IC_50_ (nM)
KB	Cervical carcinoma	4.0±0.5
HONE-1	Nasopharyngeal carcinoma	4.6±0.8
H460	Non-small-cell lung carcinoma	5.8±1.0
MKN45	Gastric carcinoma	6.0±2.3
TSGH	Gastric carcinoma	10.7±3.2
HT-29	Colorectal carcinoma	12.5±3.7
MCF-7	Breast carcinoma	17.89±1.4

Each value represents the mean ± S.D. of at least three independent experiments.

**Table 2:** Antitumor efficacy of MPT0B214 against KB-derived cell lines with different resistance phenotypes.

			IC_50_ (nM)	
KB-derived cell lines	Resistant Type	B214	Vincristine	Paclitaxel
KB	Parental	2.9±0.5	0.7±0.2	4.5±1.4
KB-S15	P-gp170/MDR	5.2±1.1	2.0±0.6	135±5.8
KB-Vin10	P-gp170/MDR	5.2±0.8	95.1±6.8	15,580±682
KB-7D	MRP	5.3±0.9	1.5±0.5	8.2±0.7

All resistant cell lines are maintained in drug-free medium for 3 days before seeding for growth inhibition assay. Each Value represents the mean ± S.D. of at least three independent experiments.

### MPT0B214 Inhibits the Polymerization of Microtubules in KB and KB-derived MDR Cell Lines

**Figure 2:**
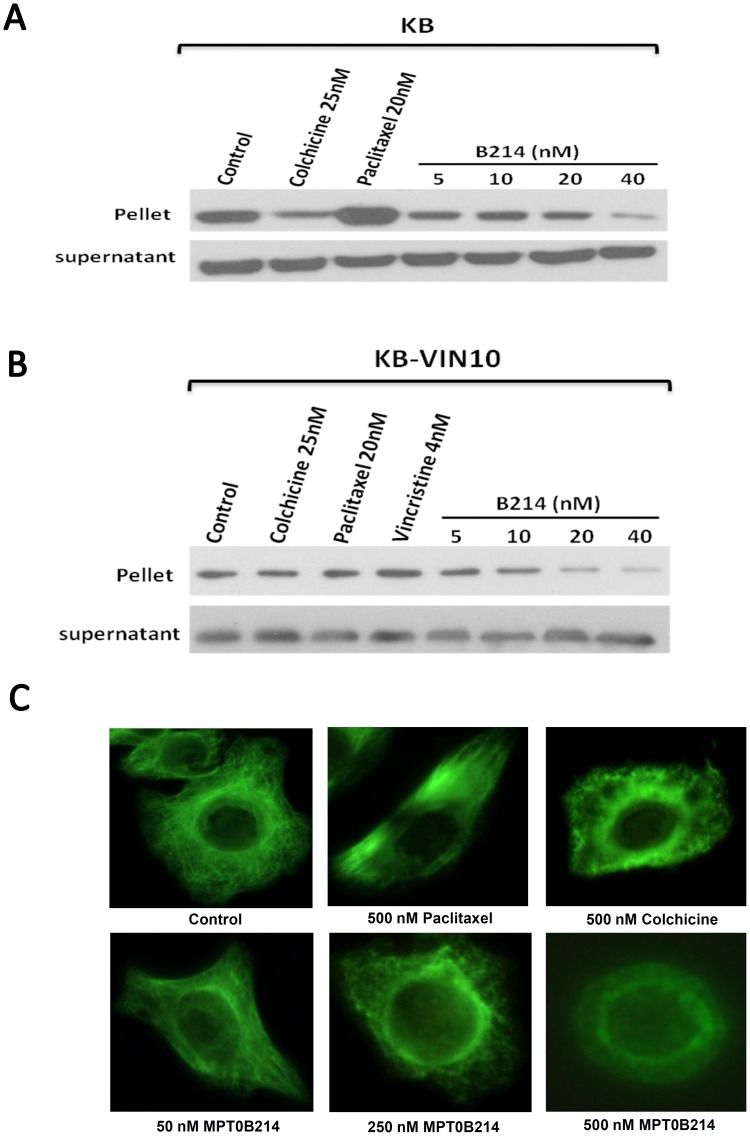
MPT0B214 inhibits microtubule assembly *in vivo*. (A) KB and (B) KB-VIN10 cells were treated with test drugs or the same volume of DMSO in PBS as the control. After 24 h of incubation, cells were lysed in a lysis buffer. Cell lysates were centrifuged to separate polymerized microtubules (pellet) from tubulin dimmers (supernatant), as described in “Material and Methods”. After gel electrophoresis and transfer to the nitrocellulose membrane, α-tubulin was visualized by western blot analysis. (C) The effect of MPT0B214 on the organizations of the cellular microtubule network. Cells were treated with 500 nM colchicine, 500 nM paclitaxel, and MPT0B214 at concentrations of 50 nM, 250 nM, and 500 nM for 6 h. After incubation, cells were harvested and fixed with a fix solution. Fixed cells were reacted with monoclonal anti-α-tubulin antibody at room temperature for 2 h. After treatment with an FITC-conjugated secondary antibody, the cellular microtubules were observed using an Olympus-BX50 fluorescence microscope.

We examined the effects of MPT0B214 on microtubule assembly at the cellular level in KB and KB-VIN10 cells. Figure 2A shows that inhibition of the microtubule assembly in the presence of colchicine and the polymerization of tubulin was increased in paclitaxel-treated cells. Similar to the effect of colchicine, MPT0B214 also inhibited tubulin polymerization in a concentration-dependent manner. We further examined the effect of MPT0B214 on microtubule polymerization in KB-VIN10 cells overexpressing P-gp170/MDR and MRP. As shown in Fig. 2B, an effect on the microtubules after treatment with colchicine, paclitaxel or vincristine was not seen in KB- VIN10 cells. However, MPT0B214 still can inhibit microtubule polymerization in a concentration-dependent manner in KB-VIN10 cells.

We examined the effects of MPT0B214 on the microtubule network by using immunofluorescence microscopy. KB cells were treated with various anti-mitotic agents for 6 h. The microtubule network exhibited a normal arrangement and organization in KB cells without treatment. Cells treated with paclitaxel caused dense microtubules scattering throughout the cytoplasm and the formation of long thick microtubule bundles. Colchicine significantly promoted cellular microtubule depolymerization, resulting in a destroyed and short filament-like structure. MPTB0214 treatment resulted in similar findings to those of colchicine-induced microtubule changes. When treatment with 500 nM of MPT0B214, we observed an almost complete loss of microtubules with only a diffuse stain visible throughout the cytoplasm (Fig. 2C). Our results show that MPT0B214 inhibit microtubule assembly in a concentration-dependent manner.

### MPT0B214 Induces Cell Cycle Arrest in G_2_-M Phase and Changes on Expression and Phosphorylation Status of G_2_-M Regulatory Proteins in KB Cells

**Figure 3:**
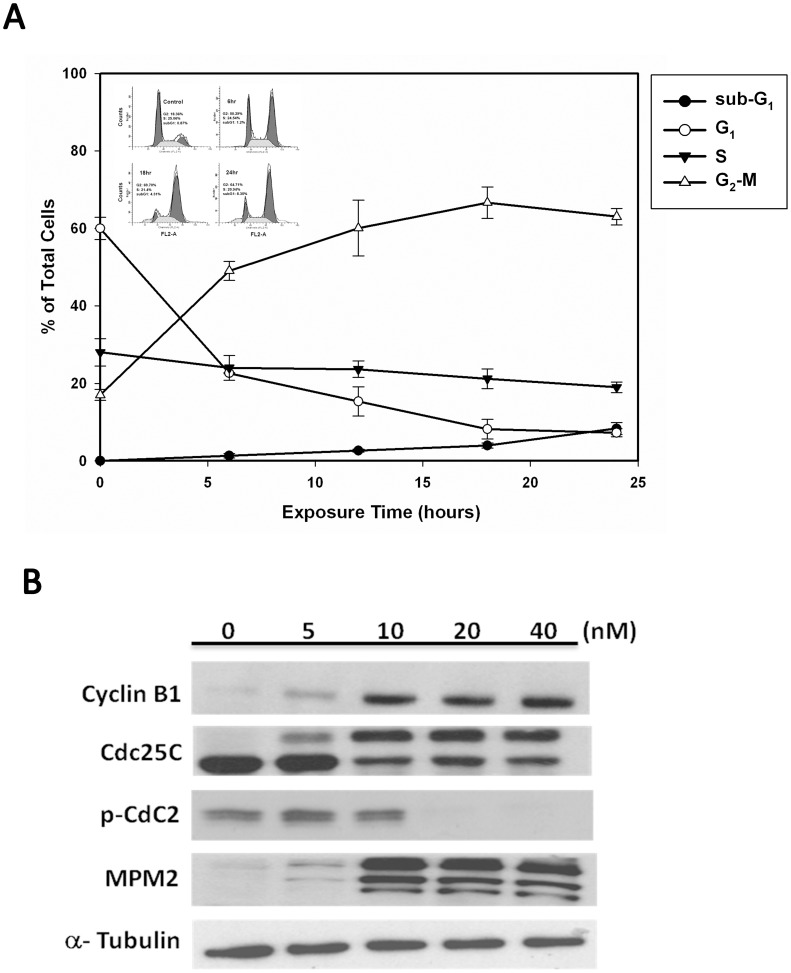
MPT0B214 induces G_2_-M phase arrest and change in the expressed and phosphorylated status of G_2_-M regulators, followed by cell apoptosis in KB cells. (A) The time effect of MPT0B214 on cell cycle distribution in KB cells (inserted figure: DNA histogram). Cells were treated with 10 nM MPT0B214 for different time points and analyzed for PI-stained DNA content by flow cytometry (B) Change in the expressed phosphorylated status of G_2_-M regulators under treatment. Cells are harvested after MPT0B214 treatment for 24 h, and the cell extract was prepared and loaded on SDS-PAGE. After electrophoresis, proteins were transferred to blots and probed with cyclin B1, Cdc25C, pCdc2, MPM2, and an internal control, α-tubulin antibodies.

The effect of MPT0B214 on cell cycle progression of KB cells was examined by flow cytometry. The results (Fig. 3A) showed that MPT0B214 leads to a time-dependent accumulation of KB cells in the G_2_-M phase, with concomitant losses in the G_0_–G_1_ phase. No obvious change in the S-phase was observed. MPT0B214 treatment-related G_2_-M arrest started after 6 h of exposure and reached a plateau at 18 h. The sub-G_1_ population with a characteristic hypodiploid DNA content peak, which is indicative of apoptosis, increased marginally. Next, we examined the relationship between MPT0B214-induced G_2_-M phase arrest and alterations in G_2_-M regulatory protein expression. As shown in Fig. 3B, MPT0B214 caused concentration-dependent increases in cyclin B1 and Cdc25C up-shifting phosphorylation. In contrast, phosphorylated cyclin-dependent kinase, pCdc2, was downregulated in a concentration-dependent manner. We further examined the status of phosphorylated polypeptides found only in mitotic cells using MPM-2 antibody. After 24 h of treatment by MPT0B214, significant elevation of the level of MPM-2 phosphoeitopes at a concentration of 10 nM was noted.

### MPT0B214 Inhibits Microtubule Polymerization by Binding to the Colchicine-binding Site

**Figure 4:**
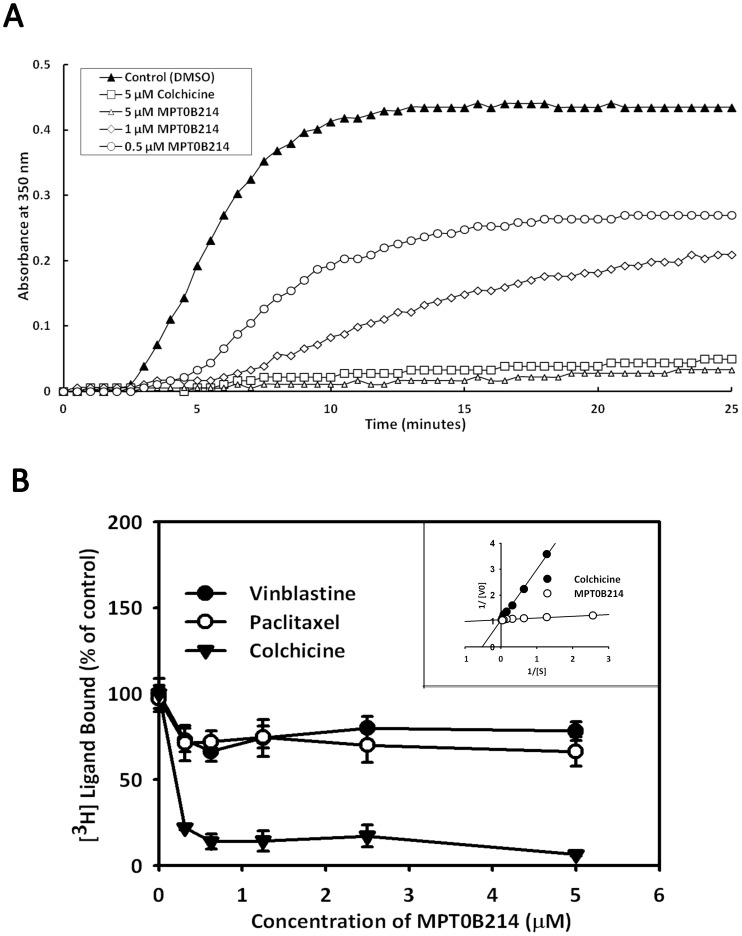
MPT0B214 inhibits microtubule polymerization *in vitro* and binds to the colchicine-binding site of tubulin. (A) MPT0B214 inhibits microtubule assembly in vitro. MAP-rich tubulin was incubated at 37°C in the absence (control, ▴) or presence of colchicines (5 μM, □) and MPT0B214 (0.5 μM, ○; 1 μM, ◊; 5 μM, △). Absorbance at 350 nM was measured every 30 s for 25 min. (B) [^3^H] ligand bound curve. Tubulin was incubated with the radio-labeled ligand at 37°C, and the amount of radio-ligand bound was determined using scintillation proximity assay beads. [^3^H] colchicine (▾), [^3^H] paclitaxel (○), [^3^H] vinblastine (•) competition binding curve, respectively. Each data point, the mean ± S.D. Double-reciprocal plot showed that MPT0B214 is a competitive inhibitor of colchicine binding to tubulin, colchicine (•), and MPT0B214 (○) (insert).

To determine the effect of MPT0B214 on the microtubule assembly *in vitro*, we incubated purified, unpolymerized, microtubule-associated protein-rich tubulin with various concentrations of MPT0B214 and measured the level of tubulin polymerization. The results showed that tubulin polymerization is inhibited in a concentration-dependent manner of MPT0B214, with an IC_50_ of 0.61±0.08 μM (Fig. 4A). MPT0B214 disrupted tubulin assembly nearly entirely at 5 μM. Because MPT0B214 inhibits microtubule assembly similarly as to colchicine and vinblastine, competition-binding scintillation proximity assay was performed to determine if MPT0B214 binds to the same intracellular target as those compounds. The results showed that MPT0B214 did not reduce the specific SPA counts, which were stimulated by conjugating the biotin–labeled tubulin with [^3^H] paclitaxel or [^3^H] vinblastine, but it competed with [^3^H] colchicine binding to the tubulin. The inhibition constant of MPT0B214 (*K_i_*, 0.04 μM) was much lower than that of colchicine (*K_i_*, 1.29 μM) (Fig. 4B and insert).

### MPT0B214 Changes the Mitochondria Membrane Potential and Causes Bcl-2 Phosphorylation Followed by Cytochrome c Translocation and Apoptosis

**Figure 5:**
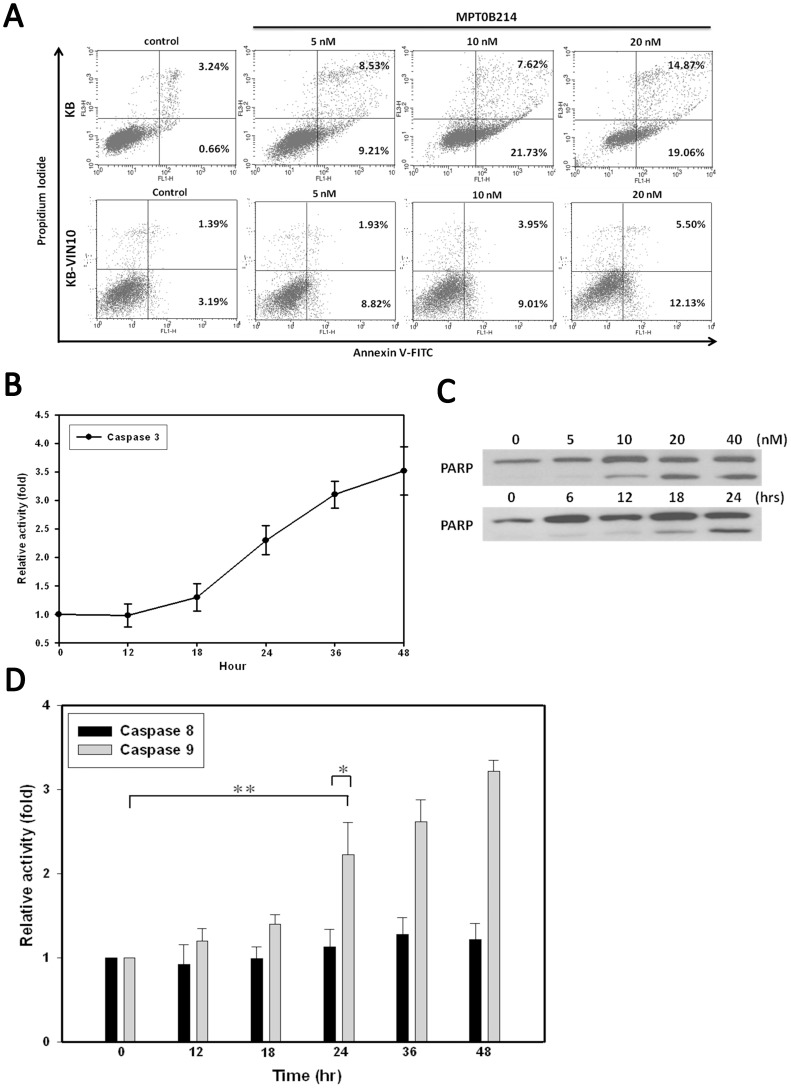
MPT0B214 induces apoptosis mainly through the activation of caspase 9. (A) Annexin V-fluorescein (FL-1)/PI (FL-2) double staining of KB and KB-VIN10 cells exposed to different concentrations of MPT0B214 for 24 h. Left bottom, annexin V^−/^PI^−^ represents viable cells; right bottom, annexin V^+^/PI^−^ indicates early apoptotic cells; right top, Annexin V^+^/PI^+^ represents late apoptotic cells. (B) Time increase in caspase 3 activity in KB cells after MPT0B214 treatment. Cells were treated with 10 nM of MPT0B214 for the indicated time and we analyzed the activity of caspase 3 by using the fluorogenic tetrapeptide Ac-DEVD-AMC (caspase 3 substract) cleavage assay. Each point represents the mean ± S.D. (C) The effect of MPT0B214 on PARP cleavage. KB cells were treated with various concentrations of MPT0B214 for 24 h and 10 nM MPT0B214 for different periods (left and right). (D) Time-course analysis of caspase enzyme activities induced by MPT0B214. The enzyme activities were determined after treatment with 10 nM of MPT0B214 for the indicated time by using LEHD-AFC (caspase 9 substract) and IEHD-AFC (caspase 8 substract) fluorogenic substracts. The relative activity unit is expressed as the ratio of the experimental sample to control. Each point represents the mean ± S.D. *p<0.05, which is significantly different compared with the control group by *t* test analysis. **p<0.05, which is significantly different compared to caspase 8 activity with caspase 9 activity by *t* test analysis.

To assess the apoptotic population of MPT0B214-treated cells, we performed annexin V-PI double staining to monitor the phosphatidylserine exposure, a signal of early apoptosis. The populations of early apoptotic cells (annexin V^+^/PI^−^) and late apoptotic cells (annexin V^+^/PI^+^) increased in a concentration-dependent manner in both KB and KB-VIN10 cells (Fig. 5A). The results showed that MPT0B214-induced cell death was attributable to apoptosis, in both KB and multidrug-resistant cell lines.

Because MPT0B214 led to the activation of apoptosis, we therefore investigated the effects of MPT0B214 on caspases. Of the many caspases involved, we first examined the effects of MPT0B214 on caspase 3. The enzymatic activity of caspase 3 rose in a time-dependent manner, beginning with 18 h of exposure of MPT0B214 and reaching a maximum by 48 h of treatment of MPT0B214 (Fig. 5B). Furthermore, cleavage of PARP, a well-known substrate for caspase 3 cleavage that forms an 85-kDa fragment [19], appeared after the concentration of MPT0B214 exceeded 10 nM and was concomitant with the increase in caspase 3 activity at 18 h (Fig. 5C). To understand how caspase 3 is activated by MPT0B214, we examined the effects of this compound on the activity of caspase 8 and caspase 9. As shown in Fig. 5D, the activity of caspase 9 elevated significantly after 12 h of exposure (1.15-fold compared with the control group) and reached a maximum of 3.2-fold induction by 48 h of MPT0B214 treatment. No significant activation of caspase 8 activity was noted. Furthermore, no changes of the expression levels Fas/Fas-L in MPT0B214-treated cells were seen (data not shown).

**Figure 6:**
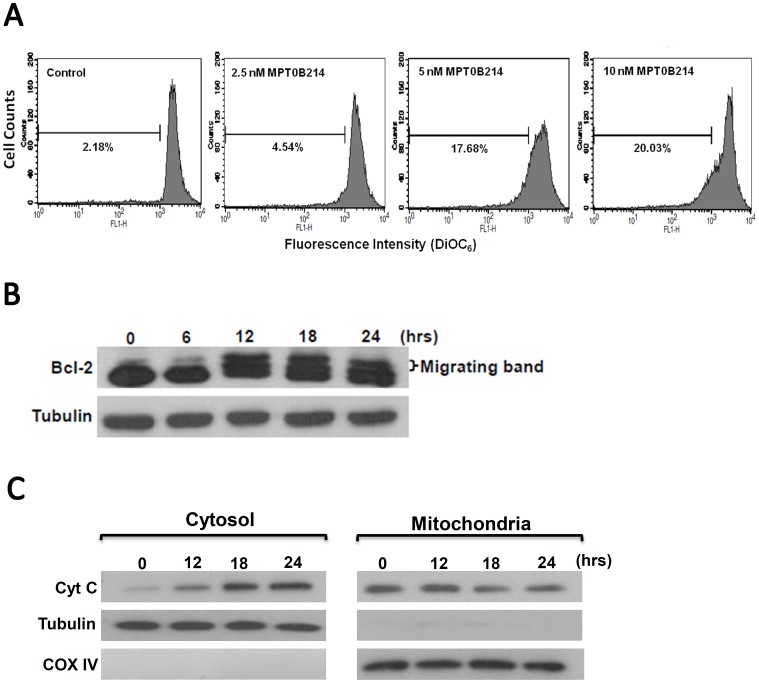
MPT0B214 induces changes in the mitochondrial membrane potential and initiates Bcl-2 phosphorylation, followed by cytochrome *c* translocation.

To confirm the involvement of mitochondria-initiated intrinsic pathways, we examined the mitochondrial membrane permeability and Bcl-2 protein phosphorylation. Ampholytic cationic fluorochrome DiOC_6_ was used to monitor changes in mitochondrial membrane potential [20]. The percentage of cells with reduced DiOC_6_ fluorescence was 20.03% after treatment with 10 nM MPT0B214 for 24 h (Fig. 6A). Cellular uptake of fluorochrome DiOC_6_ showed a significant reduction after treatment with various concentrations of MPT0B214, indicating a loss of mitochondrial membrane potential in a concentration-dependent manner. The Bcl-2 protein located in the outer mitochondrial membrane is necessary for maintaining mitochondrial integrity. Furthermore, its phosphorylation is a common characteristic of destabilized mitochondria [21], [22]. Figure 6B shows that the appearance of the characteristically slower migrating band of Bcl-2, began to be detected after treatment with 10 nM MPT0B214 for 12 h. Moreover, MPT0B214 induced a time-dependent translocation of cytochrome c from the mitochondria to the cytosol (Fig. 6C).

## Discussion

Targeting microtubules as a chemotherapeutic target has been used successfully in clinic fields, such as taxanes and the vinca alkaloids. However, although they have been extensively used in the treatment of various malignancies, numerous problems remain to be solved [23]. Drug resistance often develops after prolonged treatment with the same agents, where the mechanism may be the expression of multidrug resistance related protein. To overcome these hurdles, agents that either bind to or act differently on microtubules are being continuously developed. We have identified MPT0B214 as a new antimitotic compound that suppresses microtubule polymerization, triggers cell apoptosis, and overcomes drug resistance.

MPT0B214 is a novel synthetically compound derived from the backbone of 5-amino-2-aroylquinolines that exhibits broad-spectrum anti-proliferative abilities at the nanomolar range against human nasopharyngeal, lung, gastric, and breast and colorectal cancer cells *invitro* (table 1), but not in normal lung tissue. Drug resistance is a known problem in the use of clinically available drugs in the treatment of malignant diseases, which is limited by their being substrates of efflux pumps, P-gp170/MDR, and MRP. Notably, MPT0B214 exerts similar potency to 3 KB-derived MDR/MRP-positive cell lines *in vitro*, which are resistant to paclitaxel, vincristine, and etoposide, respectively (table 2). These results show that MPT0B214 probably is not a substrate of either P-gp170/MDR or MRP.

To clarify the molecular targets of MPT0B214, we first examined if it targets microtubules. Our data showed that MPT0B214 strongly inhibits microtubule assembly in KB and KB-VIN10 cells (Fig. 2A and 2B), disrupts intracellular microtubule networks in intact cells, as shown in the immunocytochemistry study (Fig. 2C), and depolymerizes microtubules by binding to the colchicine-binding sites of tubulin (Fig. 4). Similar to other microtubule-interacting agents, MPT0B214 arrests the growth of cancer cells at the G_2_-M phase, as indicated by flow cytometry analysis (Fig. 2A). In eukaryotic cells, cyclin B and Cdc2 kinase regulate the onset of M phase [24], [25]. Previous studies have shown that the activation of Cdc2 kinase is controlled by an accumulation of cyclin B and de-phosphorylation of Cdc2. The activation of Cdc2 requires the hyper-phosphorylation of Cdc25C [26], [27], [28]. Treatment with MPT0B214 not only directly contributes to disrupting microtubules in cells, but also induces accumulation of cyclin B1 and reduces phospho-Cdc2 kinase and phosphorylation Cdc25C. In addition, a concentration-dependent increase in the expression of phosphoepitopes is recognized by MPM-2 antibodies, which are mitosis-specific markers and are only detected in mitotic cells (Fig. 2B). These changes of protein expression are consistent with cell cycle arrest in mitosis [29].

Apoptosis induced by anti-mitotic agents is known to be related to alternations of cellular signaling pathways. In addition to the effect of MPT0B214 on the cell cycle, it also provokes apoptosis, as shown by the findings of annexin V/PI in KB and KB-VIN10 cells. To investigate the involvement of apoptosis pathways in MPT0B214-mediated cytotoxicity, we assessed the caspase cascades. First, we examined the activity of caspase 3, which increases in a time-dependent manner after cells are treated with MPT0B214 (Fig. 5B). A cleaved-form of PARP, a well-known substrate for caspase 3, appears and significantly increases in a concentration- and time-dependent manner (Fig. 5C). To investigate how caspase 3 is activated, we assessed the effect of this compound on the activity of caspase 8 and caspase 9. In contrast to zero to mild activation of caspase 8 activity, MPT0B214 activates caspase 9 activity in a concentration-dependent manner (Fig. 5D). Moreover, because caspase 8 is one of the most apical caspases on Fas/FasL mediated apoptosis, neither the expression level of the Fas ligand nor that of the Fas protein is changed by MPT0B214 treatment in our experiments (data not shown). These findings show that a Fas/FasL-triggered extrinsic apoptotic pathway is not involved in MPT0B214-induced apoptosis.

To confirm that mitochondria-mediated intrinsic pathways are involved in MPT0B214-mediated apoptosis, we further monitored the changes of mitochondrial membrane potential, the status of cytochrome C translocation and Bcl-2 protein. Our data showed a loss of mitochondrial membrane potential in cells treated with MPT0B214 (Fig. 6A). Furthermore, the collapse of mitochondrial membrane potential results in the efflux of certain proteins and small molecules, such as cytochrome c. When cytosolic cytochrome c meets procaspase 9, the apoptosome is formed, which subsequently stimulates proteolytic activation of caspase 3 [30]. Figure 6C shows that MPT0B214 induces a time-dependent effect on cytochrome *c* translocation from the mitochondria into the cytosol. Evidence has shown that the Bcl-2 family plays a central role in the control of apoptosis through inhibiting programmed cell death and apoptosis [22], [31], [32]. Once Bcl-2 phosphorylates, it loses its protective effect to maintain the mitochondrial membrane potential and permeability. Microtubule inhibitors such as vincristine, colchicine, paclitaxel and docetaxel induce cell cycle arrest, followed by phosphorylation and inactivation of Bcl-2, and then leading to apoptotic cell death [31], . In Fig. 6B, MPT0B214 could induce slow-migrating band shift of Bcl-2 in a time-dependent manner, which is likely to be phosphorylation [10], [11]. Thus, these results showed that MPT0B214-induced apoptosis is associated with mitochondria-mediated intrinsic pathways.

It is interesting that KB-VIN10 cells are blunter to undergo apoptosis over a range of MPT0B214 concentrations than KB cells, but close IC50 values between these 2 cell lines (Figure 5A and table 2). Possibilities of this discrepancy were listed below. First, KB VIN10 cells were KB-derived MDR-positive cell line by long-term selection with vincristine 10 nM. In addition well-known P-gp170/MDR, other genetic differences between these two cell lines could exist, such as mutations in the tubulin gene. Second, different expression in tubulin isotypes may be one of possible mechanisms and further studies are needed to validate these inferences.

In conclusion, we demonstrated that MPT0B214, a synthetic aroylquinoline compound with strong activity against microtubule assembly, exhibits broad-spectrum anticancer properties against various solid tumor cells. Notably, MPT0B214 may overcome drug resistance in cells overexpressing P-gp 170/or MRP and displays no cross resistance to known existing microtubule inhibitors, including vinca alkaloids and taxanes. Therefore, MPT0B214 is an effective and promising new tubulin-binding compound and is worth further exploration.
